# Mechanically Activated Transition from Linear Viscoelasticity to Yielding: Correlation-Based Unification

**DOI:** 10.3390/polym17192665

**Published:** 2025-10-01

**Authors:** Maxim S. Arzhakov, Irina G. Panova, Aleksandr A. Kiushov, Aleksandr A. Yaroslavov

**Affiliations:** Department of Chemistry, M.V. Lomonosov Moscow State University, Leninskie Gory 1, 119991 Moscow, Russia

**Keywords:** mechanics, yielding, plastics, foams, correlation analysis, unification, prediction

## Abstract

The mechanically activated transition (MAT) from linear viscoelasticity to yielding is considered an essential part of the operational behavior of ductile materials. The MAT region is restricted by proportional limit at *σ_0_* and *ε_0_* and the yield point at *σ_y_* and *ε_y_*, or, in terms of this paper, $E_{0}=\sigma_{0}/\varepsilon_{0}$ and *ε_0_* and $E_{y}=\sigma_{y}/\varepsilon_{y}$ and *ε_y_*, respectively. This stage precedes yielding and controls the parameters of the yield point. For bulk plastic (co)polymers and cellular polymeric foams, the quantitative correlations between $E_{0}$, *ε_0_*, $E_{y},$ and *ε_y_* were determined. The ratios E0Ey=1.55±0.15 and εyε0=2.1±0.2 were specified as yielding criteria. For all the samples studied, their mechanical response within the MAT region was unified in terms of master curve constructed via re-calculation of the experimental “stress–strain” diagrams in the reduced coordinates $\frac{\mathrm{lg}\;E-\mathrm{lg}\;E_{0}}{\mathrm{lg}\;E_{0}-\mathrm{lg}\;E_{y}}=f\left(\frac{\mathrm{lg}\;\varepsilon -\mathrm{lg}\;\varepsilon_{0}}{\mathrm{lg}\;\varepsilon_{y}-\mathrm{lg}\;\varepsilon_{0}}\right),$ where E=σ/ε and *ε* are the current modulus and strain, respectively. To generalize these regularities found for bulk plastics and foams, our earlier experimental results concerning the rheology of soil-based pastes and data from the literature concerning the computer simulation of plastic deformation were invoked. Master curves for (1) dispersed pastes, (2) bulk plastics, (3) polymeric foams, and (4) various virtual models were shown to be in satisfactory coincidence. For the materials analyzed, this result was considered as the unification of their mechanical response within the MAT region. An algorithm for the express analysis of the mechanical response of plastic systems within the MAT region is proposed. The limitations and advances of the proposed methodological approach based on correlation studies followed by construction of master curves are outlined.

## 1. Introduction

Studies on the mechanics of ductile materials or plastics provide a fundamental background for designing products with the required and controlled operation characteristics to meet the needs of the automobile, railway, aerospace, construction, and industrial sectors [1,2].

For ductile bodies, with increasing strain, steady-state flow or yielding takes place. From an engineering standpoint, the occurrence of yielding implies a loss of load-bearing capacity of the material and construction, though their macroscopic fracture is not observed. In connection with this, parameters corresponding to the yield point (stress and strain) are associated with the ultimate operation characteristics of the material.

The mechanical behavior of the material up until the yield point is reached involves the initial region of linear viscoelasticity. Further deformation results in a deviation from the linear viscoelastic response, followed by macroscopic yielding. The transition from linear viscoelasticity to yielding is considered a mechanically activated process. This mechanically activated transition (MAT) occurs within the strain range between the linear viscoelastic strain interval and the yield point. In other words, for plastics, the MAT region is an essential part of the material’s operational behavior. Studies on the structural and mechanical regularities of this phenomenon are of prime importance from both fundamental and applied viewpoints.

For deeper insight into the mechanics of ductile solids, a variety of methodologies—from experiment-based phenomenology to profound theoretical analysis and mathematical processing based on particular models and mechanisms—have been proposed [3,4,5,6,7,8,9,10,11,12,13,14,15,16,17,18,19,20,21,22,23]. The main trends are associated with approaches characterized by a predictive nature and based on the usage of well-defined experimental characteristics.

In our recent work [24], the rheology of virgin and modified soil-based pastes was discussed in terms of correlations between the values of (1) the initial storage modulus $G_{0}^{'}$, (2) the storage modulus corresponding to yield points $G_{y}^{'}$, (3) strain *γ_0_*—the upper strain limit of linear viscoelasticity, and (4) strain *γ_y_* corresponding to the yield point. The pairs ($G_{0}^{'}$*, γ_0_*) and ($G_{y}^{'}$*, γ_y_*) represent the lower and upper limit of the MAT region, respectively.

Note that, recently, correlation analysis has been widely used in research across a variety of social disciplines, including the humanities, natural sciences, medicine, and business. This analytical and statistical methodology is associated with investigating the phenomenological relationships between two or more variables [13,14,15,16,17]. In the majority of cases, the causation of the phenomenon is beyond the scope of the discussion. In mechanics, correlation studies are focused on establishing stable and reliable “property–property”, “structure–property”, and “structure–composition–property” relationships.

The above correlations allowed us to construct a master “storage modulus–strain” diagram [24] for the dispersed materials, characterized by their different origins, structures, rheological properties, etc. From a fundamental viewpoint, this master curve demonstrates that the rheology of dispersed bodies obeys general regularity, regardless of their specific nature, structure, and physicochemical modification. This regularity is associated with the strict “pathway” of mechanical response until the yield point is reached. Note that this range of strain involves the interval of linear viscoelasticity and the MAT region. In practice, this master curve enabled prediction and express analysis of the rheological behavior of dispersed soil-based pastes using only one experimentally measured parameter—$G_{0}^{'}$.

The results obtained were used as the methodological foundation for the unified analysis of the rheology of the dispersed materials. This methodology involves “property–property” correlation studies followed by the unification of the mechanical behavior of the dispersed pastes in terms of the master curve. This approach is based on analysis and examination of the direct experimental data without employing theoretical models and mechanisms.

In this paper, the proposed methodology was extended to a unified description of the mechanical behavior of solid bulk plastics and cellular polymeric foams. A comparative analysis of our own data and data from the literature concerning the mechanical response of dispersed bodies and plastic solids was carried out. To generalize this approach, data from the literature on computer simulation and modeling of plastic deformation were invoked. The limitations and prospects of this methodological approach based on correlation studies followed by the construction of master curves are outlined.

## 2. Materials and Methods

Commercial poly(vinyl chloride) (PVS) and polystyrene (PS) (ChemRaw, Moscow, Russia) were used.

Poly(methyl methacrylate) (PMMA), PMMA plasticized with dibutyl phthalate (DBPh), and random copolymers of methyl methacrylate (MMA) with butyl methacrylate (BMA), octyl methacrylate (OMA), lauryl methacrylate (LMA), and methacrylic acid (MAA) were synthesized via bulk (co)polymerization.

Prior to (co)polymerization, the above monomers (Sigma-Aldrich, Burlington, MA, USA) were distilled in a vacuum under a nitrogen flow. Lauroyl peroxide (Sigma-Aldrich), used as the initiator, was purified by recrystallization from ethanol. The content of initiator in the polymerizing systems was 5 × 10^–3^ mol/L.

The monomer feed compositions were as follows: for MMA/BMA—80/20, 70/30, and 50/50; for MMA/OMA—95/5, 90/10, and 80/20; for MMA/LMA—95/5, 90/10, and 80/20; and for MMA/MAA—75/25. Before (co)polymerization, monomer mixtures containing the initiator were deoxygenated by repeated freezing–defreezing at a pressure of 10^–2^ mmHg.

(Co)polymerization was carried out in sealed glass tubes at 60 °C. To provide complete conversion, at the final stage of the reaction, the temperature was increased to a temperature which exceeded glass transition temperature of the corresponding (co)polymers by 10–15 °C.

Polyurethane foams (PUFs) with densities of 30, 45, and 60 kg/m^3^ were prepared by sputtering using the pilot setup Interskol 5H200 (Interskol, Moscow, Russia) under a pressure of 90 atm and at 22 °C. The density of each test sample was measured at room temperature by hydrostatic weighting.

Two-component composition involved commercial bifunctional aromatic polyester VP-3317 (Vladipur, Vladimir, Russia) and commercial aliphatic polyester VP-3700 (Vladipur, Vladimir, Russia) with a functionality of 2.3 (component A), and commercial polyisocyanate Wannate PM-200 (Wanhua Chemical, Yantai, China) (component B). The isocyanate index of compositions used—that is, the molar ratio between reactive isocyanate and alcohol groups [I] = [NCO]/[OH]—varied from 0.8 to 1.45. To align the viscosities of components A and B, oligomeric polyatomic alcohols were added to component A.

Freon/water and methylal/water systems were used as foaming agents. The density of the final PUFs was controlled by varying the content of foaming agent.

Mechanical testing of the samples was carried out using Insrtron 3400 (Instron, Buckinghamshire, UK).

Bulk PS, PVC, PMMA, and copolymer cylindrical samples with a diameter of 10 mm and a height of 10 mm were uniaxially compressed at 20, 40, 50, and 70 °C with strain rates of 10^−2^, 10^−3^, 10^−4^, and 10^−5^ s^−1^. PUF samples with a diameter of 20 mm and a height of 20 mm were uniaxially compressed at 20 °C with a strain rate of 10^−3^ s^−1^.

Young’s modulus was estimated as the slope of the linear initial portion of the “stress–strain” diagram with an accuracy of ±7%. The strain and stress corresponding to the proportional limit and yield point were estimated with an accuracy of ±4% and ±5%, respectively.

## 3. Results and Discussions

**Figure 1:**
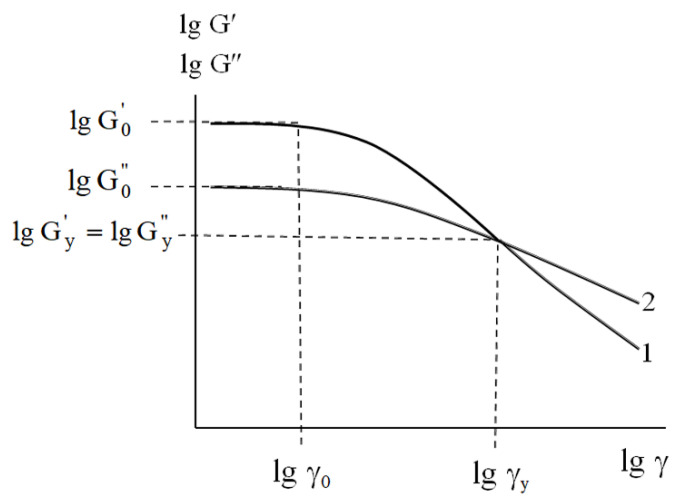
Typical strain dependencies of storage modulus G’ (1) and loss modulus G’’ (2) for dispersed soil-based samples. See details in text.

In an earlier study [24], the rheological behavior of virgin and modified Chernozem, turf-podzolic soil, kaolinite, montmorillonite, and quartz sand pastes was studied using dynamic loading in amplitude sweep mode. The experimental results were discussed in terms of the complex modulus $G^{*}=G^{'}+\;G^{”}i$, where G'=τ'γ*,* G″=τ″γ*, γ, τ′*, and *τ″* are the storage modulus, loss modulus, strain, and real and imaginary parts of complex stress, respectively. The typical dependences of *lg*$\;G^{'}$ and $\mathrm{lg}\;G^{”}$ on strain *lg* γ are shown in Figure 1.

The typical results of the dynamic testing (Figure 1) allowed us to recognize the following strain intervals and basic mechanical parameters which describe the rheological behavior of the dispersed soil-based pastes.

Within the strain interval $\mathrm{lg}\;\gamma <\mathrm{lg}\gamma_{0}$, the linear viscoelastic response of the samples is characterized by constant values of both the initial storage modulus $\mathrm{lg}\;G_{0}^{'}$ and the initial loss modulus $\mathrm{lg}\;G_{0}^{”}$. The constancy of these parameters implies high stability of the material’s initial structure—that is, a physical network of mineral particles—under loading conditions [25,26].

At $\mathrm{lg}\;\gamma >\mathrm{lg}\gamma_{0}$, under strain growth, a decrease in both $\mathrm{lg}\;G^{'}$ and $\mathrm{lg}\;G^{”}$ is associated with the partial microscopic rupture of inter-particle contact, followed by the complete macroscopic destruction of the abovementioned physical network. For the dispersed systems, macroscopic physical network destruction is considered to represent “yielding” [27,28,29]. Yielding manifests as a cross-over point at the yield strain $\mathrm{lg}\gamma_{y}\;$ when $\mathrm{lg}\;G_{y}^{'}=\mathrm{lg}G_{y}^{”}$ (Figure 1). Obviously, at $\mathrm{lg}\;\gamma <\mathrm{lg}\gamma_{y}$, the elastic response prevails ($\mathrm{lg}\;G^{'}>\mathrm{lg}G^{”}$), while at $\mathrm{lg}\;\gamma >\mathrm{lg}\gamma_{y}$, well-pronounced fluidity occurs ($\mathrm{lg}\;G^{'}<\mathrm{lg}G^{”}$).

From this standpoint, the experimental parameters $\mathrm{lg}\;G_{0}^{'}$*,* $\mathrm{lg}\;G_{y}^{'}$*,* $\mathrm{lg}\;\gamma_{0}$, and $\mathrm{lg}\;\gamma_{y}$ completely describe the rheological behavior of the materials. Note that the pair $\mathrm{lg}\;G_{y}^{'}$ and $\mathrm{lg}\;\gamma_{y}$ represents the ultimate operation characteristics of the dispersed bodies when a complete loss of load resistance takes place.

**Figure 2:**
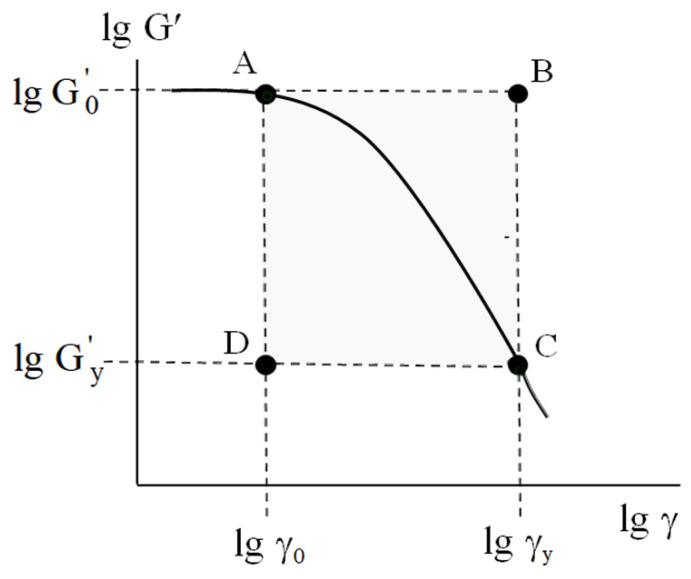
Schematic representation of MAT region for typical strain dependence of storage modulus G′ for dispersed soil-based pastes. See details in text.

From both fundamental and applied viewpoints, the portion of the rheological curve $\mathrm{lg}\;G^{'}=f\left(\mathrm{lg}\;\gamma \right)$ (curve 1, Figure 1) in the strain interval $\mathrm{lg}\;\gamma_{0}<\mathrm{lg}\gamma <\mathrm{lg}\gamma_{y}$ is of the most interest. This range corresponds to the mechanically activated transition (MAT) from linear viscoelasticity to yielding, accompanied by the complete destruction of the structural network. In Figure 2, which details the strain dependence of the storage modulus, the MAT region is schematically represented as a highlighted area.

Obviously, the coordinates of corners A, B, C, and D of the rectangular MAT box are set by the couples of parameters ($\mathrm{lg}\;G_{0}^{'}$ and $\mathrm{lg}\;\gamma_{0}$), ($\mathrm{lg}\;G_{0}^{'}$ and $\mathrm{lg}\;\gamma_{y}$), ($\mathrm{lg}\;G_{y}^{'}$ and $\mathrm{lg}\;\gamma_{y}$), and ($\mathrm{lg}\;G_{y}^{'}$ and $\mathrm{lg}\;\gamma_{0}$), respectively. In other words, the size of the MAT box is defined as $\left(\mathrm{lg}\;G_{0}^{'}-\mathrm{lg}\;G_{y}^{'}\right)\times \left(\mathrm{lg}\gamma_{y}-\mathrm{lg}\gamma_{0}\right)$.

For the soil-based pastes studied (more than 50 virgin and modified samples), linear correlations between (A) $\mathrm{lg}\;G_{0}^{'}$ and $\mathrm{lg}\;\gamma_{0}$, (B) $\mathrm{lg}\;G_{0}^{'}$ and $\mathrm{lg}\;\gamma_{y}$, and (C) $\mathrm{lg}\;G_{0}^{'}$ and $\mathrm{lg}\;G_{y}^{'}$ were found [24]. These “property–property” correlations provide evidence that the rheological parameters $\mathrm{lg}\;G_{y}^{'}$, $\mathrm{lg}\;\gamma_{0}$, and $\mathrm{lg}\;\gamma_{y}$ of the dispersed samples are strictly determined by the value of initial storage modulus $\mathrm{lg}\;G_{0}^{'}$. The higher the $\mathrm{lg}\;G_{0}^{'}$ value is, the higher the $\mathrm{lg}\;G_{y}^{'}$ value is and the lower the $\mathrm{lg}\;\gamma_{0}$ and $\mathrm{lg}\;\gamma_{y}$ values are.

Hence, experimental measurement of the $\mathrm{lg}\;G_{0}^{'}$ value provides an estimation of the $\mathrm{lg}\;\gamma_{0}$, $\mathrm{lg}\;G_{y}^{'}$, and $\mathrm{lg}\;\gamma_{y}$ values without direct experimental testing. Note that the value of $\mathrm{lg}\;\gamma_{0}$ is considered to represent the upper strain limit of viscoelasticity. The values of $\mathrm{lg}\;G_{y}^{'}$ and $\mathrm{lg}\;\gamma_{y}$ correspond to yielding—that is, the upper limit of the material’s operation.

**Figure 3:**
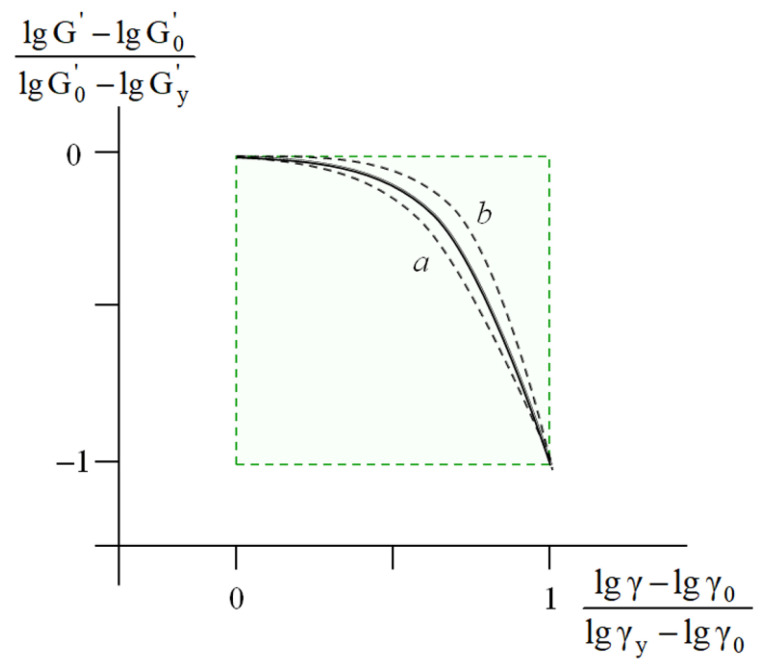
Master curve for dynamic testing of both virgin and modified soil-based samples. Dash lines a and b show scatter of re-calculated experimental curves [24].

These correlations allowed us to propose a procedure for the unification of the rheological behavior of the soil-based pastes within the MAT box [24]. This approach involved the treatment of the experimental curves *lg G′ = f (lg γ)* in the dimensionless reduced coordinates $\frac{\mathrm{lg}\;G^{'}-\mathrm{lg}\;G_{0}^{'}}{\mathrm{lg}\;G_{y}^{'}-\mathrm{lg}\;G_{0}^{'}}=f\left(\frac{\mathrm{lg}\gamma -\mathrm{lg}\gamma_{0}}{\mathrm{lg}\gamma_{y}-\mathrm{lg}\gamma_{0}}\right)$, and the construction of unified master curve for all the samples studied (Figure 3).

Hence, the results obtained allow one to conclude that, in the wide range of applied strain, the rheology of the dispersed soil-based pastes is controlled by the initial values of the storage modulus $G_{0}^{'}$.

From a practical viewpoint, the construction of the master curve (Figure 3) enabled prediction and express analysis of the rheological behavior of both the virgin and modified soil-based pastes using only one experimentally measured parameter—$G_{0}^{'}$. The algorithm and verification of this procedure are detailed in [24].

In this case, the following question arises: Are these results unique to the rheology of dispersed pastes, or they can be applied to describe the mechanical behavior of solid plastics? To clarify this problem, the following analysis of the mechanics of the plastic bodies was carried out.

**Figure 4:**
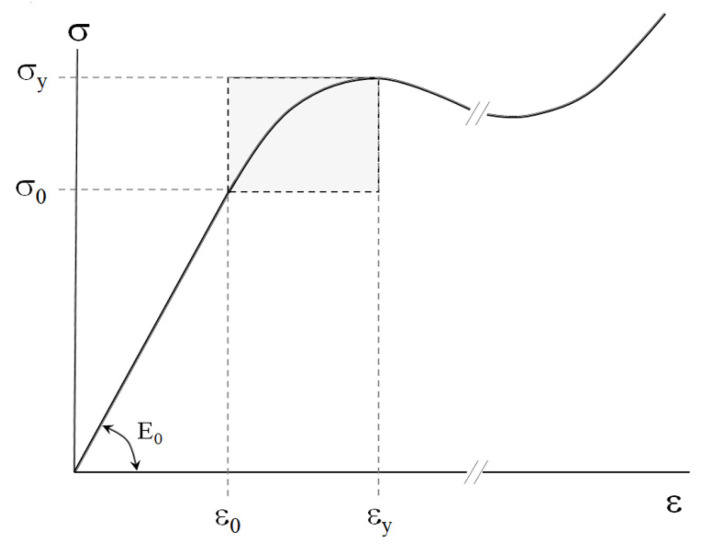
Typical “stress–strain” diagram for plastics. See details in text.

To study mechanical behavior of plastics, the deformation under regimes of shearing, uniaxial drawing, uniaxial compression, torsion, bending, etc., of samples with a fixed strain rate $\dot{\varepsilon }=d\varepsilon /dt$ is widely used [1,2,7]. The results of mechanical testing are usually represented as the dependence of stress *σ* on strain *ε* (“stress–strain” diagram) (Figure 4).

Note that, for plastics, typical stress–strain behavior (Figure 4) is characterized by the proportional limit at *σ_0_* and *ε_0_* and the yield point at *σ_y_* and *ε_y_*.

At $\varepsilon \leq \varepsilon_{0}$ and $\sigma \leq \sigma_{0}$, the linear viscoelastic response of the plastics obeys Hooke’s law and is characterized by Young’s modulus $E_{0}=\sigma_{0}/\varepsilon_{0}$.

At $\varepsilon >\varepsilon_{y}$, macroscopic yielding or mechanically activated steady-state fluidity of the material takes place. Similarly to the rheological behavior of the abovementioned dispersed bodies, mechanically activated transition (MAT) from linear viscoelasticity to yielding at $\varepsilon_{0}\leq \varepsilon \leq \varepsilon_{y}$ occurs (highlighted area in Figure 4).

For the virgin soils and the soils plasticized with dibutyl phthalate (DBPh) poly(methyl methacrylate) (PMMA), polystyrene (PS), and poly(vinyl chloride) (PVC), as well as the copolymers of methyl methacrylate (MMA) with butyl methacrylate (BMA), octyl methacrylate (OMA), lauryl methacrylate (LMA), and methacrylic acid (MAA), studied in this work, detailed examination of their stress–strain behavior under uniaxial compression at the specified deformation temperature *T_def_* and strain rate $\dot{\varepsilon }$ was carried out (Table 1).

**Figure 5:**
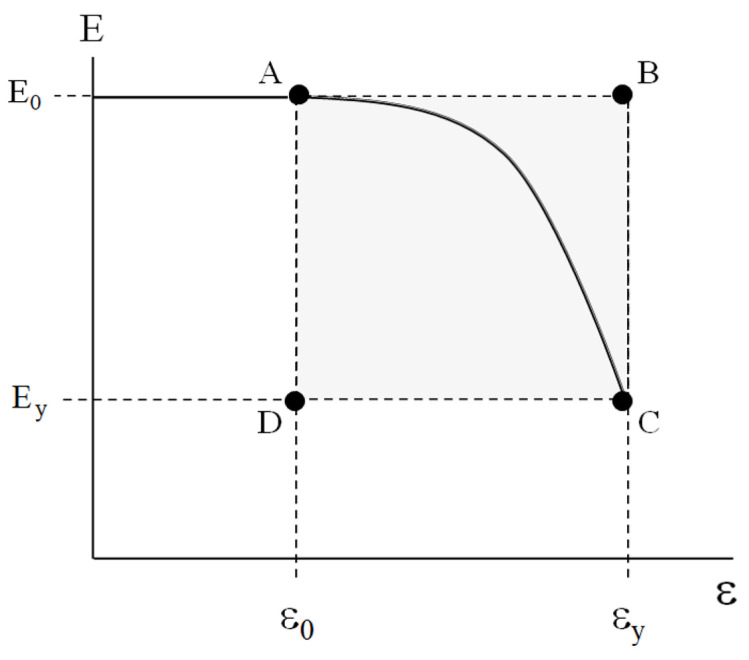
Typical strain dependence of current modulus for plastics. See details in text.

For comparative analysis of the dispersed bodies’ rheology and the plasticity of the bulk polymers, the stress–strain diagrams of the plastic samples were re-calculated in the coordinates $E=f\left(\varepsilon \right)$, where E=σ/ε is the current modulus of the material. The typical strain dependence of the current modulus is shown in Figure 5.

Obviously, at $\varepsilon \leq \varepsilon_{0}$, the initial modulus *E_0_* is constant. Within the MAT box (highlighted area in Figure 5), the current modulus *E* falls and reaches the value of $E_{y}=\sigma_{y}/\varepsilon_{y}$. Corners A, B, C, and D of the rectangular MAT box correspond to the couples of parameters (*E_0_* and *ε_0_*), (*E_0_* and *ε_y_*), (*E_y_* and *ε_y_*), and (*E_y_* and *ε_0_*), respectively. The size of the MAT box is defined as *(E_0_ − E_y_) × (ε_y_ − ε_0_)*.

**Figure 6:**
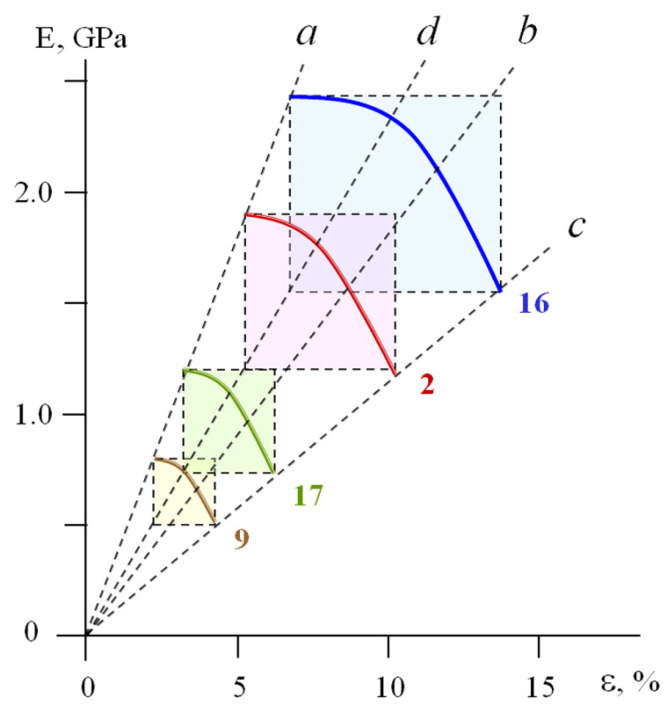
MAT boxes for samples 2, 9, 16, and 17 (Table 1). Uniaxial compression at 20 °C with strain rate $\dot{\varepsilon }=1.7\times 10^{-4}s^{-1}$.

For samples 2, 9, 16, and 17 (Table 1), their MAT boxes are shown in Figure 6. Lines a, b, c, and d go through corners A, B, C, and D of these rectangular MAT boxes, respectively, and imply the linear dependences $E_{0}=f\left(\varepsilon_{0}\right)$, $E_{0}=f\left(\varepsilon_{y}\right)$, $E_{y}=f\left(\varepsilon_{y}\right)$, and $E_{y}=f\left(\varepsilon_{0}\right)$.

For an individual polymer, the values of *E_0_*, *ε_0_, E_y_*, and *ε_y_* decrease as a result of plasticization, a decrease in the strain rate $\dot{\varepsilon }$, and an increase in the deformation temperature *T_def_.*

In our earlier work [7,30,31], for the stress–strain behavior of carbon chains, hetero-chains, and hetero-cyclic polymers, the following ratios of the mechanical parameters were found:

$$E_{0}\frac{\varepsilon_{y}}{\sigma_{y}}=1.6\pm 0.2$$

or

(1) E0Ey=1.6 ±0.2

and

(2) εyε0=2.0±0.2

**Figure 7:**
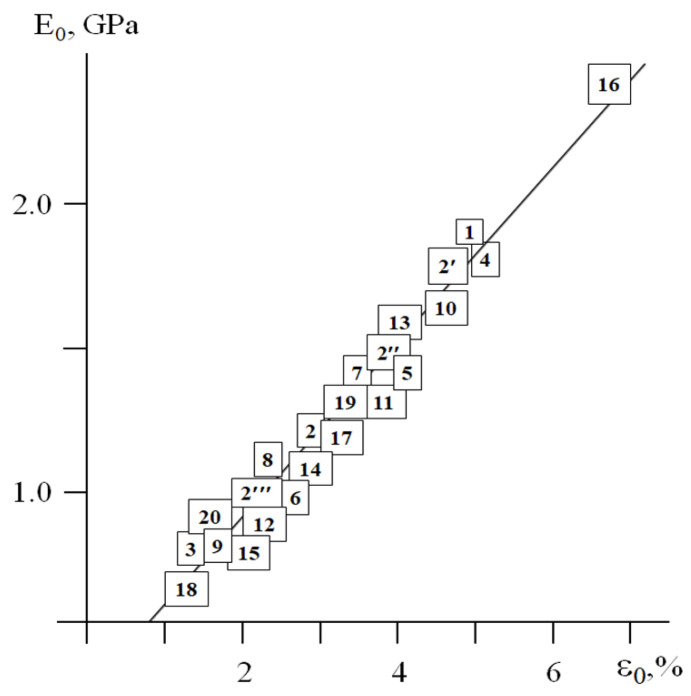
The dependence of *E_0_* on *ε_0_*. The numbers labeling the experimental data correspond to the sample numbers in Table 1. Pearson’s r is 0.96012.

Expressions (1) and (2) demonstrate that the dimensions of the MAT box *(E_0_ − E_y_) × (ε_y_ − ε_0_)* are controlled by only two parameters—*E_0_* and *ε_0_*_._ For the plastic samples listed in Table 1, Figure 7 shows the correlation between these two characteristics.

Note that the plastics (Table 1) with the different chemical structure tested at a fixed *T_def_* and $\dot{\varepsilon }$ (samples 1, 7–17, 19), the plasticized polymers (4–6) and individual polymers tested at a fixed $\dot{\varepsilon }$ and different *T_def_* (1–3, 17–20), and the polymers tested at a fixed *T_def_* and different $\dot{\varepsilon }$ (2′, 2″, 2, 2′″) satisfy this dependence. In other words, this $E_{0}=f\left(\varepsilon_{0}\right)$ correlation takes into account the influence of the chemical and physicochemical modification of the polymers, as well as the influence of the time–temperature regime of deformation on the mechanical behavior of the plastics.

Expressions (1) and (2) and Figure 7 demonstrate that, for the plastics, the size of their MAT boxes is set by the only one parameter: the initial modulus *E_0_*. Experimental measurement of this parameter allows one to estimate (1) the value of *ε_0_* using the linear correlation shown in Figure 7, and (2) the values of *E_y_* and *ε_y_* using Expressions (1) and (2)—that is, the complete set of characteristics that describes the plasticity of a polymer.

Similarly to the bulk plastics, rigid polymeric foams demonstrate well-pronounced plasticity, with the yield point characterized by the yield stress *σ_y_* and the yield strain *ε_y_* [32,33,34,35,36,37,38,39].

As for bulk plastics, the mechanical parameters (*E_0_, σ_y_, ε_y_, σ_0_,* and *ε_0_*) of the foams depend on the chemical structure of the polymer, as well as on the time–temperature regime of the deformation. With a decrease in the deformation temperature *T_def_* and an increase in the strain rate $\dot{\varepsilon }$, linear growth in these charasteristics is observed.

Note that the mechanical behavior of the polymeric foams is mainly controlled by their density. A decrease in the density results in a decrease in the above mechanical parameters of the foams. Additional factors which affect the mechanical properties of foams are associated with (1) the morphology of the cellular structure, (2) the configuration and size of both the cells and intercellular walls, (3) the nature of gas which fills the cells (air, nitrogen, carbon dioxide, freon, etc.), and (4) the foam production technology used.

Hence, as compared with bulk plastics, a description of the mechanical behavior of polymeric foams is considered a more complicated problem when the above factors are taken into account.

In this work, to advance in this scientific direction, mechanical testing of a series of PUF samples prepared by sputtering was carried out. The chemical structure and composition of the samples were varied by the usage of aromatic and aliphatic polyesters, as well as by variation of isocyanate index [I] = [NCO]/[OH] from 0.8 to 1.45. The density of the foams was varied from 30 to 60 kg/m^3^ by variation of the content of two types of foaming agents (freon/water and methylal/water systems).

To gain a deeper insight into the mechanical behavior of these materials, stress–strain diagrams from the literature for PUFs with different chemical structures—polyvinylchloride, polystyrene, and polyethylene foams produced via casting, extrusion, and spraying—were analyzed. These samples, which had a density ranging from 30 to 980 kg/m^3^, were uniaxially compressed at –54, 20, and 74 °C with strain rates of 10^–4^, 10^–3^, and 10^–2^ s^–1^ [32,35,36,39].

The treatment of our own data and data from the literature concerning the mechanical behavior of the above foams (more than one hundred stress–strain diagrams) revealed the following correlations:

(3) E0Ey=1.55±0.15

and

(4) εyε0=2.5±0.25

As for the bulk plastics and foams, a linear correlation between *E_0_* and *ε_0_* was found (Pearson’s r is 0.95674).

The above correlations (Expressions (1)–(4), Figure 7) were based on the treatment of experimental “stress–strain” diagrams for both bulk and cellular polymeric materials.

To expand this approach to other types of ductile bodies (for example, metal glasses), systematic and profound long-term studies are required. In this paper, to verify the validity of the correlation analysis, the results of the computer simulation of plastic deformation were invoked. Note that the computer-simulated mechanical response reflects regularities of plasticity that are general for materials with different natures, origins, structures, etc.

Computer-simulated “stress–strain” diagrams characterized by a well-pronounced MAT region and a yield point were considered. Analysis involved the following models, theoretical approaches, and deformation regimes: shearing of binary systems of disks [40]; molecular–dynamic simulation of low-temperature uniaxial compression and extension [9]; uniaxial extension and shearing in the framework of the atomistic–continuum model [41]; uniaxial compression using a free-volume-based constitutive model [11]; and damage plastic modeling of uniaxial tension [19]. The simulated “stress–strain” diagrams were re-calculated in $E=f\left(\varepsilon \right)$ coordinates and the values of *E_0_, E_y_, ε_0_*, and *ε_y_* were estimated. For these parameters, the following correlations were found:

(5) E0Ey=1.5±0.15

and

(6) εyε0=1.7±0.25

Let us summarize the set of the results based on the treatment of more than 200 experimental stress–strain diagrams for (1) bulk plastics, (2) cellular polymeric foams, and (3) virtual models under shearing, uniaxial drawing, and compression with a fixed strain rate.

For all the samples studied, deformation behavior is characterized by the mechanically activated transition (MAT) from linear viscoelasticity to macroscopic fluidity or yielding. The dimensions of the MAT region are written as $\left(E_{0}-E_{y}\right)\times \left(\varepsilon_{y}-\varepsilon_{0}\right)$. Note that the yield point with coordinates $\left(E_{y},\;\varepsilon_{y}\right)$ corresponds to the ultimate operation characteristic when a complete loss of load bearing capacity takes place.

For both the bulk plastics and the cellular foams, as well as for the virtual models, the experimental and computer-simulated deformation behavior obeys similar regularities associated with the comparable values of the ratios E0Ey and εyε0 (Table 2).

These ratios can be considered the criteria for yielding. Macroscopic yielding takes place when the current strain *ε* approaches the yield strain *ε_y_ (ε → ε_y_)* and the current modulus *E* approaches the yield modulus *E_y_ (E → E_y_)*. Taking into account the average values of the corresponding ratios (Table 2), yielding occurs when εε0→2.1±0.2 and E0E→1.55±0.15.

The above correlations demonstrate that the dimensions of the MAT regions are controlled by the only one parameter—Young’s modulus *E_0_*. The experimentally measured value of *E_0_* specifies unambiguously (1) the value of *ε_0_*—that is, the upper strain limit of the linear viscoelasticity region (Figure 7)**—** and (2) the values of $E_{y}\;and\;\varepsilon_{y}$ (Table 2), which define the yield point of the material. In other words, the value of *E_0_* allows one to estimate the abscissa of point A (*ε_0_*) and (2) the position of point C $\left(E_{y},\varepsilon_{y}\right)$ (Figure 5) without direct experimental testing.

However, these results provide no information concerning the path of the system from point A to point C within the MAT box (Figure 5). To reveal the regularities of this trajectory, let us invoke the procedure proposed in [24] to unify the rheological behavior of both the virgin and modified soil-based pastes.

For the bulk plastics, the above correlations of the basic mechanical parameters (Figure 7, Table 2) imply the geometrical similarity of the MAT boxes (Figure 6). Their dimensions are controlled by a change in the chemical nature and composition of the materials, as well as the time–temperature regime of their deformation. Obviously, these geometrically similar rectangles within a portion of the deformation curves can be transformed into each other as follows.

**Figure 8:**
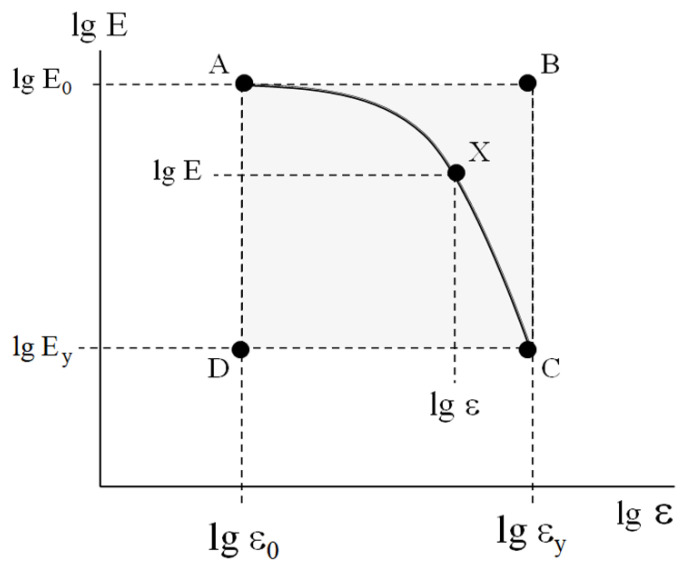
A schematic representation of the MAT box.

To compare the results concerning the mechanical behavior of the bulk plastics and the rheology of the soil-based pastes [24], let us represent the MAT box (Figure 5) on a logarithmic scale (Figure 8). Note that point A restricts the interval of linear viscoelasticity and point C corresponds to the yield point.

**Figure 9:**
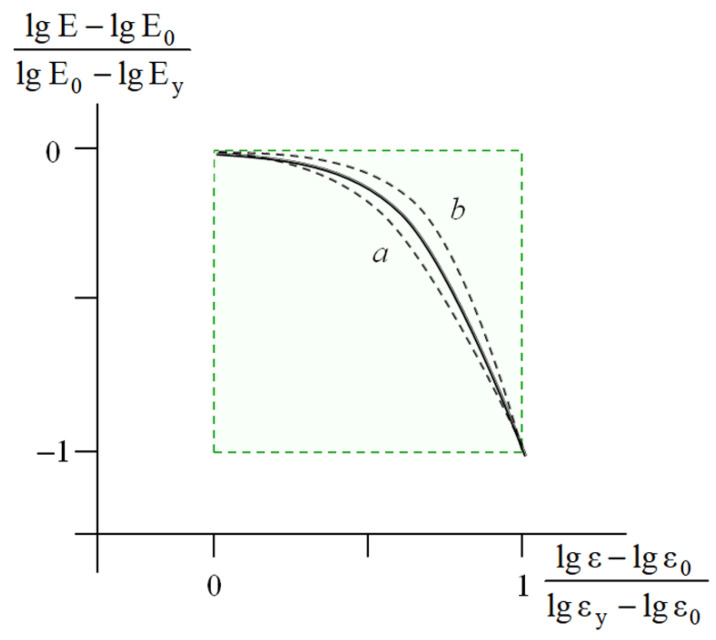
The master curve for the deformation of the bulk plastics under a constant strain rate. Dashed lines *a* and *b* show the scatter of the re-calculated experimental curves.

In this case, for any point X (Figure 8) with the coordinates $\left(\mathrm{lg}\;E,\;\mathrm{lg}\;\varepsilon \right)$, the deviation of the value of the current modulus *lg E* from the initial value *lg E_0_*, namely, $\left(\mathrm{lg}\;E-\mathrm{lg}E_{0}\right)$ was normalized by the height of the MAT box—$\left(\mathrm{lg}\;E_{0}-\mathrm{lg}E_{y}\right)$. The abscissa of the MAT box was re-calculated in a similar way. The resulting master curve in the coordinates $\frac{\mathrm{lg}\;E-\mathrm{lg}\;E_{0}}{\mathrm{lg}\;E_{0}-\mathrm{lg}\;E_{y}}=f\left(\frac{\mathrm{lg}\varepsilon -\mathrm{lg}\varepsilon_{0}}{\mathrm{lg}\varepsilon_{y}-\mathrm{lg}\varepsilon_{0}}\right)$ is shown in Figure 9.

**Figure 10:**
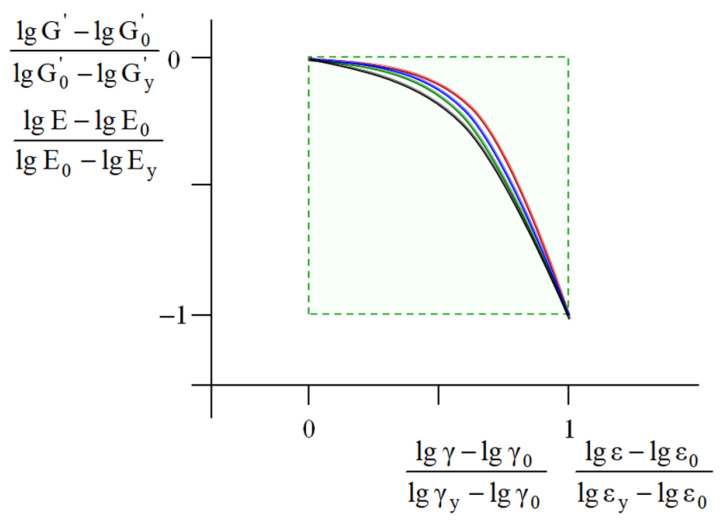
Master curves for the dispersed soil-based samples (red line) in the coordinates $\frac{\mathrm{lg}\;G^{'}-\mathrm{lg}\;G_{0}^{'}}{\mathrm{lg}\;G_{y}^{'}-\mathrm{lg}\;G_{0}^{'}}=f\left(\frac{\mathrm{lg}\gamma -\mathrm{lg}\gamma_{0}}{\mathrm{lg}\gamma_{y}-\mathrm{lg}\gamma_{0}}\right)$, and for the bulk plastics, polymeric foams, and virtual models (blue, green, and black lines, respectively) in the coordinates $\frac{\mathrm{lg}\;E-\mathrm{lg}\;E_{0}}{\mathrm{lg}\;E_{0}-\mathrm{lg}\;E_{y}}=f\left(\frac{\mathrm{lg}\varepsilon -\mathrm{lg}\varepsilon_{0}}{\mathrm{lg}\varepsilon_{y}-\mathrm{lg}\varepsilon_{0}}\right)$.

Similar master curves were constructed for the uniaxially compressed polymeric foams and computer-simulated virtual models. For the physical and virtual systems studied in this work, as well as for the soil-based pastes [24], the master curves are in good agreement (Figure 10).

The obtained results demonstrate that within the MAT region, the mechanical response of the materials obeys strict regularity, independent of their origin, their chemical structure, their morphology, their physicochemical modification, and the time–temperature regime of the deformation.

## 4. Conclusions

For (1) bulk ductile (co)polymers, (2) polymeric foams with different cellular morphology, and (3) various virtual models, the mechanically activated transition (MAT) from linear viscoelasticity to yielding was analyzed.

The MAT region is restricted by the proportional limit, with the coordinates *E_0_* and *ε_0_*, and the yield point, with the coordinates *E_y_* and *ε_y_* (Figure 5). Note that, in practice, the yield point is considered the ultimate operational characteristic of plastics when a complete loss of load resistance takes place. The size of the MAT box *(E_0_ − E_y_) × (ε_y_ − ε_0_)* depends on the chemical structure, composition, and morphology of the materials, as well as on the time–temperature regime of testing.

The significance of studies in the MAT region is associated with the fact that this phenomenon is an essential part of the deformation of ductile bodies. This event precedes yielding, and thus controls the operational behavior of the material.

For the mechanical characteristics (*E_0_, ε_0_, E_y_* and *ε_y_*) restricting the MAT region, quantitative correlations were found—$E_{0}/E_{y}=1.55\pm 0.15$ and $\varepsilon_{y}/\varepsilon_{0}=2.1\pm 0.2$ (Table 2).

These correlations imply the following criteria for yielding: yielding occurs when the current modulus *E* falls by 1.55±0.15 as compared with Young’s modulus *E_0_*, and when the current strain *ε* reaches a value of (2.1 ± 0.2) × ε_0_. Note that the values of *E_0_* and *ε_0_* are in linear correlation (Figure 7).

The obtained results demonstrate the geometrical similarity of the rectangular MAT boxes (shown for bulk plastics, Figure 6). These geometrically similar rectangles can be transformed into each other via re-calculation of the experimental curves $E=f\left(\varepsilon \right)$ in the dimensionless reduced coordinates $\frac{\mathrm{lg}\;E-\mathrm{lg}\;E_{0}}{\mathrm{lg}\;E_{0}-\mathrm{lg}\;E_{y}}=f\left(\frac{\mathrm{lg}\varepsilon -\mathrm{lg}\varepsilon_{0}}{\mathrm{lg}\varepsilon_{y}-\mathrm{lg}\varepsilon_{0}}\right)$.

This procedure resulted in the construction of the master curve shown in Figure 9 for the bulk plastics. Similar master curves were constructed for the deformation of polymeric foams and computer-simulated deformation of various virtual models.

The satisfactory coincidence of the master curves for the dispersed soil-based pastes [24] and the materials studied in this work indicate that their mechanical response within the MAT region satisfies the strictly defined pathway (Figure 10).

Obviously, the abovementioned physical bodies and virtual objects are characterized by a different nature, structure, and origin; their only common feature is well-pronounced plasticity or yielding. In connection with this, they can be denoted as “plastic systems”. These systems are associated with sustainable correlations between basic mechanical characteristics (Table 2, Figure 7), as well as with a strictly defined trajectory of the mechanical response within the MAT region (Figure 10). Note that this unification is based only on the analysis of more than 300 experimental and computer simulated results, with no involvement of theoretical models or deformation mechanisms.

The above analysis of the mechanical behavior of the plastic systems allowed us to conclude that, for these systems, the mechanical response up until the yield point is strictly controlled only by the initial storage and Young’s modulus. These key characteristics specify (i) the upper strain limit of the linear viscoelasticity region $\varepsilon_{0}$ and *γ_0_*, (ii) the parameters of the yield point—the pairs ${(E}_{y}{,\varepsilon }_{y})$ and ($G_{y}^{'}$, γ_y_), and (iii) the ratios E0Ey and εyε0 as the criteria for yielding.

The initial modulus can be considered a macroscopic manifestation of the initial state of the plastic system. Obviously, for the materials studied (dispersed bodies, bulk plastics, porous foams), as well as for the virtual models, their initial state is characterized by their different structural organization. However, the initial structure unambiguously sets (i) the pathway of the plastic system within the MAT region and (ii) the parameters of the yield point, that is, the upper operation limit. Note that, for the plastic systems, the similarity between their mechanical trajectories (Figure 10) does not imply similarity in the mechanically stimulated structural evolution that is responsible for and/or accompanies their deformation.

As mentioned above, from the applied standpoint, the construction of master curves (Figure 3) enabled express analysis of the mechanical behavior of the dispersed soil-based pastes using only one experimentally measured parameter—$G_{0}^{'}$ [24]. The good agreement of the master curves for the dispersed, bulk, and cellular materials (Figure 10) indicates that this approach can be applied to the express analysis of plastics and foams. Similarly to the algorithm detailed in [24], the procedure involves (1) experimental measurement of Young’s modulus *E_0_*; (2) estimation of the values of *ε_0_, E_y_*, and *ε_y_* using the above correlations (Figure 7, Table 2); (3) substitution of these parameters in the coordinates $\frac{\mathrm{lg}\;E-\mathrm{lg}\;E_{0}}{\mathrm{lg}\;E_{0}-\mathrm{lg}\;E_{y}}=f\left(\frac{\mathrm{lg}\varepsilon -\mathrm{lg}\varepsilon_{0}}{\mathrm{lg}\varepsilon_{y}-\mathrm{lg}\varepsilon_{0}}\right)$; (4) re-calculation of the master curve into the current value of *E* and *ε*; and (5) construction of *E*$\;=f\left(\varepsilon \right)$ dependence without a direct experiment.

Note that, at the present time, the mechanical behavior of each type of material analyzed in this work (dispersed pastes, bulk plastics, and cellular foams) is described in terms of various structural models, deformation mechanisms, and theoretical approaches [1,2,3,4,5,6,18]. Deeper insight into their plasticity mechanisms is provided by computer simulation of deformation with various virtual models [9,11,19,40,41]. In this paper, the general regularities of plasticity of these physical bodies and virtual models were attributed to the correlations of their basic mechanical parameters (Table 2) and unified master curves (Figure 10). The results obtained are characterized by their predictive nature and provide analysis of the operational behavior of ductile bodies using only one experimental parameter—the initial storage modulus (for dynamic testing) and Young’s modulus (for deformation with the fixed strain rate).

Future advancement in this scientific field requires comprehensive research that includes studies on the mechanical behavior of inorganic and metallic plastic materials, as well as theoretical justification and mathematical modeling of the abovementioned regularities.

The limitations of this methodology based on correlation studies followed by the construction of unified master curve are as follows. This approach is not applicable to labile materials for which mechanically stimulated phase and polymorphic structural transformations take place in the course of deformation. A prime example of these kinds of materials is polyethyleneterephtalate (PET). Deformation of PET samples is accompanied by crystallization caused by the orientation of the polymer chains. This factor is responsible for continuous changes in the structure of the polymer and resulting continuous changes in the mechanical response of the sample.

Obviously, for multi-phase and multi-component blends and composites, the deformation regularities are more complicated as compared with those for the polymeric materials discussed above. The mechanical response of blends and composites is controlled by the superposition of the micro-mechanical behavior of the components and the interphase boundary, synergetic effects, etc. These factors are specific and unique to a given composition and prevent a general description of the plasticity of these materials.

## Figures and Tables

**Table 1:** A list of the samples uniaxially compressed at the specified deformation temperature *T_def_* and strain rate $\dot{\varepsilon }$.

N	Sample	*T_def_*, °C	$\dot{\varepsilon }$, s^−1^
1 2 2′ 2″ 2′″ 3	PMMA PMMA PMMA PMMA PMMA PMMA	20 50 50 50 50 70	10^−4^ 10^−4^ 10^−2^ 10^−3^ 10^−5^ 10^−4^
4 5 6	PMMA, plasticized with DBPh (mole ratio) 95/5 90/10 80/20	20 20 20	10^−4^ 10^−4^ 10^−4^
7 8 9	poly(MMA-*co*-BMA) (mole ratio) 80/20 70/30 50/50	20 20 20	10^−4^ 10^−4^ 10^−4^
10 11 12	poly(MMA-*co*-OMA) (mole ratio) 95/5 90/10 80/20	20 20 20	10^−4^ 10^−4^ 10^−4^
13 14 15	poly(MMA-*co*-LMA) (mole ratio) 95/5 90/10 85/15	20 20 20	10^−4^ 10^−4^ 10^−4^
16	poly(MMA-*co*-MAA) (mole ratio) 75/25	20	10^−4^
17 18	PS PS	20 40	10^−4^ 10^−4^
19 20	PVC PVC	20 40	10^−4^ 10^−4^

**Table 2:** Correlation of the basic parameters for various systems deformed under a constant strain rate.

	Bulk Plastics	Polymeric Foams	Computer Simulation	Average Value
$\varepsilon_{y}/\varepsilon_{0}$ $E_{0}/E_{y}$	2.0 ± 0.2 1.6 ± 0.2	2.5 ± 0.25 1.55 ± 0.15	1.7 ± 0.25 1.5 ± 0.15	2.1 ± 0.2 1.55 ± 0.15

## Data Availability

Dataset available on request from the authors.
